# Hyperparasitaemia during bouts of malaria in French Guiana

**DOI:** 10.1186/1475-2875-12-20

**Published:** 2013-01-16

**Authors:** Bernard Carme, Magalie Demar

**Affiliations:** 1 Laboratory of Parasitology and Mycology, Research team EA 3593, Faculty of Medicine, University Antilles Guyane, and Centre Hospitalier de Cayenne, Rue des Flamboyants, Cayenne, BP 6006, F- 97354, French Guiana; 2 Centre d’Investigation Clinique - Epidémiologie Clinique Antilles Guyane (CIC-EC INSERM CIE 802), Cayenne General Hospital, Cayenne, French Guiana; 3 Unit of Infectious and Tropical Diseases, Cayenne Hospital, Cayenne, French Guiana

**Keywords:** Malaria, *Plasmodium falciparum*, *Plasmodium vivax*, Hyperparasitaemia, Severe malaria, French Guiana

## Abstract

**Background:**

High circulating parasite load is one of the WHO criteria for severe *falciparum* malaria. During a period of 11 years (2000–2010), the frequency of hyperparasitaemia (HP) (≥4% infected erythrocytes) during bouts of malaria due to *Plasmodium falciparum, Plasmodium vivax* and *Plasmodium malariae* in patients referred to Cayenne General Hospital (CGH) in French Guiana and the frequency of their admission to the Intensive Care Unit (ICU) were evaluated.

**Methods:**

A mean of 1,150 malaria cases were referred to the Parasitology Laboratory of CGH each year over the last decade. During this period, malaria diagnostic (microscopy) and parasitaemia evaluation have remained unchanged: determination of the parasitized erythrocytes percentage with asexual forms on thin blood smears for all cases of parasitaemia exceeding 0.1%. Patients admitted to the ICU can be counted by origin of the request for malaria testing. All the data collected retrospectively were anonymized in a standardized case report form and in database.

**Results:**

Between 2000 and 2010, 12,254 bouts of malaria were confirmed at the Parasitology Laboratory of CHG: *P. vivax*: 56.2%, *P. falciparum*: 39.5%, co-infection with both species: 3.4%, *P. malariae*: 0.9%. HP was observed in 262 cases, at a frequency of 4.9% for *P. falciparum* and only 0.041% for *P. vivax,* with no recorded cases for *P. malariae.* The need for intensive care was correlated with *P. falciparum* parasite load: 12.3% of cases for parasitaemia of 4-9%, 21.2% for parasitaemia 10-19%, 50% for parasitaemia 20-29% and 77.8% for parasitaemia ≥30% (n=9). The patient with the highest parasitaemia (75% infected erythrocytes with asexual form) presented a major concomitant lupus flare-up treated with corticoids. He survived without obvious sequelae.

**Conclusions:**

In French Guiana during bouts of malaria, HP was observed at a frequency of ~ 5% for *P. falciparum* and two orders of magnitude less frequent for *P. vivax*. HP is a severity criterion for falciparum malaria in this endemic area. However, two of the patients with HP ≥30% were not admitted to the ICU and sequel-free cure in malaria patients with 75% parasitaemia is, therefore, possible.

## Background

The regions of endemic malaria in French Guiana, which is a French Overseas territory of 235,000 inhabitants, neighboring Brazil and Suriname in the Amazonian region, had until recently the highest incidence of the disease in South America [1], but a strong decrease since 2008 [2] was noted. Disease foci are located along the large rivers bordering Suriname to the west (Maroni focus) and Brazil to the east (Oyapock focus). Three species of the parasite are present: *Plasmodium vivax* and *Plasmodium falciparum* predominate, whereas *Plasmodium malariae* is much rarer. Despite the unfavorable epidemiological conditions (isolated enclaves, substantial movement of exposed individuals, often without official papers, drug-resistant strains of *P. falciparum*), the financial and health equipment resources deployed in French Guiana have ensured that severe forms remain rare and mortality low [3].

The WHO criteria for *falciparum* severe malaria include high circulating parasite load (or hyperparasitaemia (HP)): threshold: 4% of erythrocytes infected with asexual forms [4].

During a period of 11 years, the frequency of HP during bouts of malaria in patients referred to Cayenne General Hospital (CGH) and the frequency of their admission to the Intensive Care Unit (ICU) were evaluated.

## Methods

### Malaria diagnosis and parasite count

A mean of 1,150 malaria cases were referred to the Parasitology Laboratory of CGH each year over the last decade. Cayenne is the main city of French Guiana. During this period, malaria diagnosis (microscopy) and parasitic count (PC) have remained unchanged: determination of the percentage of parasitized erythrocytes with asexual forms on thin blood smears for all cases of parasitaemia exceeding 0.1%. When PC <0.1%, semi-quantitative evaluations are carried out on thick smears. The screening sensitivity was ~ 6 Plasmodium/μl. All parasite counts were pre-treatment or the maximum PC value observed in the first 24 hours. Multiplex real-time PCR [5] were used in case of *P*. vivax HP for detecting a possible concurrent infection with *P. falciparum*.

The data were input into software (Hexaflux, Agfa), for subsequent analysis.

### Admission to the intensive care unit

CGH intensive care unit (ICU) was the only service of this type in French Guiana. The patients admitted to the ICU can be counted by origin of the request for malaria testing. Regardless of the duration of ICU hospitalization, the evaluation on admission of all patients presenting known or suspected malaria systematically included a request for malaria testing.

### Malaria treatment

The precise treatment modalities varied in French Guiana during this long period except for the treatment of severe malaria in the ICU: administration of intravenous quinine, with high dose during the first 8 hours, in combination with doxycycline or clindamycine.

### Ethical consideration

The retrospective use of anonymous patient files on the site of patient care is authorized by the French National Commission on Informatics and Liberties (CNIL). All the data collected retrospectively were anonymized in a standardized case report form and in database.

## Results

Between 2000 and 2010, 12,254 bouts of malaria were recorded: 56.2% due to *P. vivax* (n=6,887)*,* 39.5% due to *P. falciparum* (n=4,840)*,* 3.4% due to a *P. vivax/P. falciparum* mixture (diagnosis by microscopy) (n=417) and 0.9% due to *P. malariae* (n=110). HP was observed in 262 patients: *P. falciparum*: 252; *P*. *vivax*: three (presence in isolation confirmed by PCR), a combination of the two (early diagnosis by microscopy then confirmed by PCR): seven cases, with *P. falciparum* predominant in each case.

**Figure 1:**
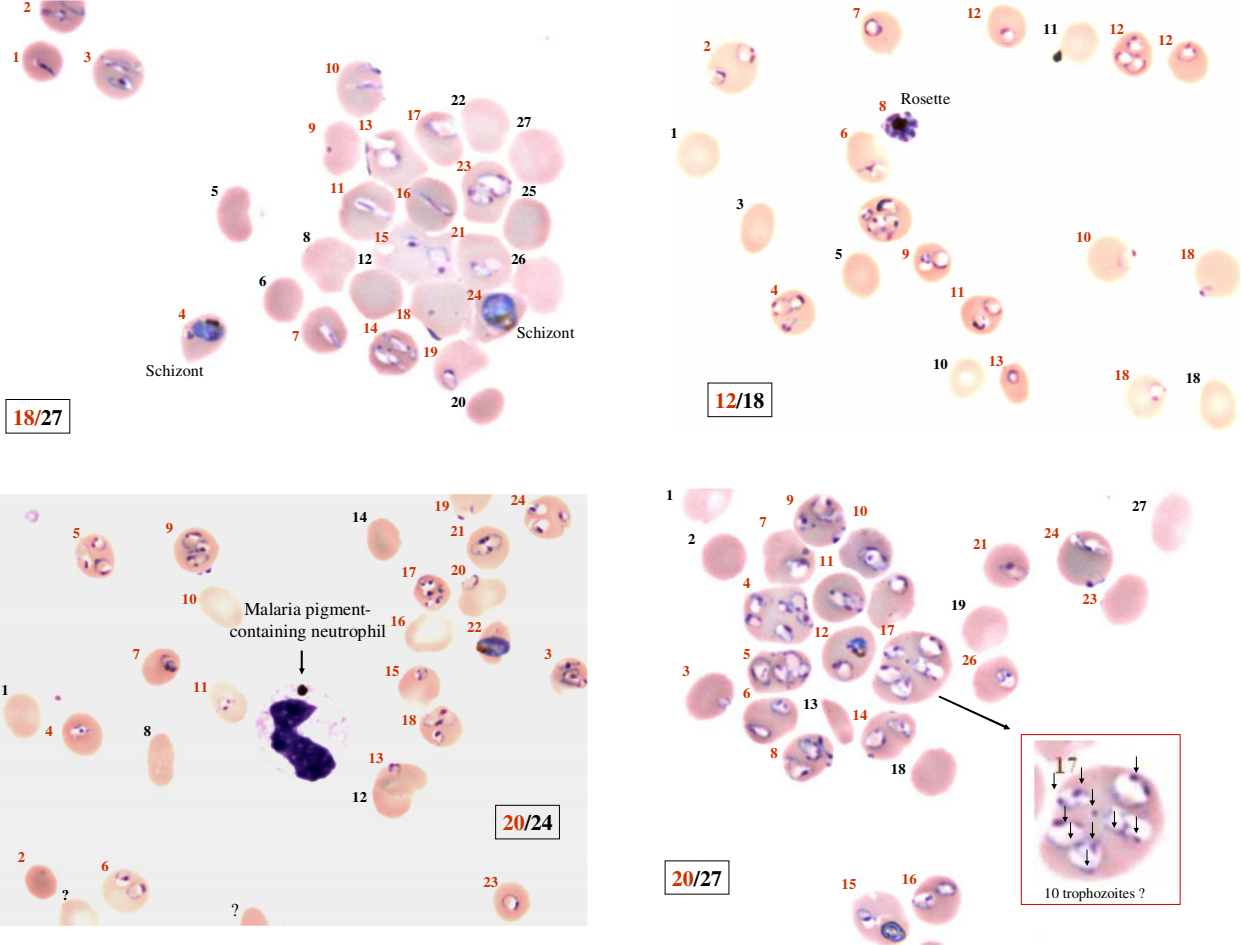
**Blood smear from a patient with hyperparasitaemia (75% parasitaemia).** This thin blood film shows strong HP (70/96 → 73% based on the photographs: *Plasmodium*-infected erythrocytes - red number- n=70, all eyrthrocytes - red and black numbers - n= 96 with young and mature schizonts (rosette) and pigmented leukocytes.

The highest parasitaemia due to asexual forms of *P. falciparum* recorded was 75%, species confirmed by PCR and precise counting on photographic prints of the blood smear (Figure 1).

**Figure 2:**
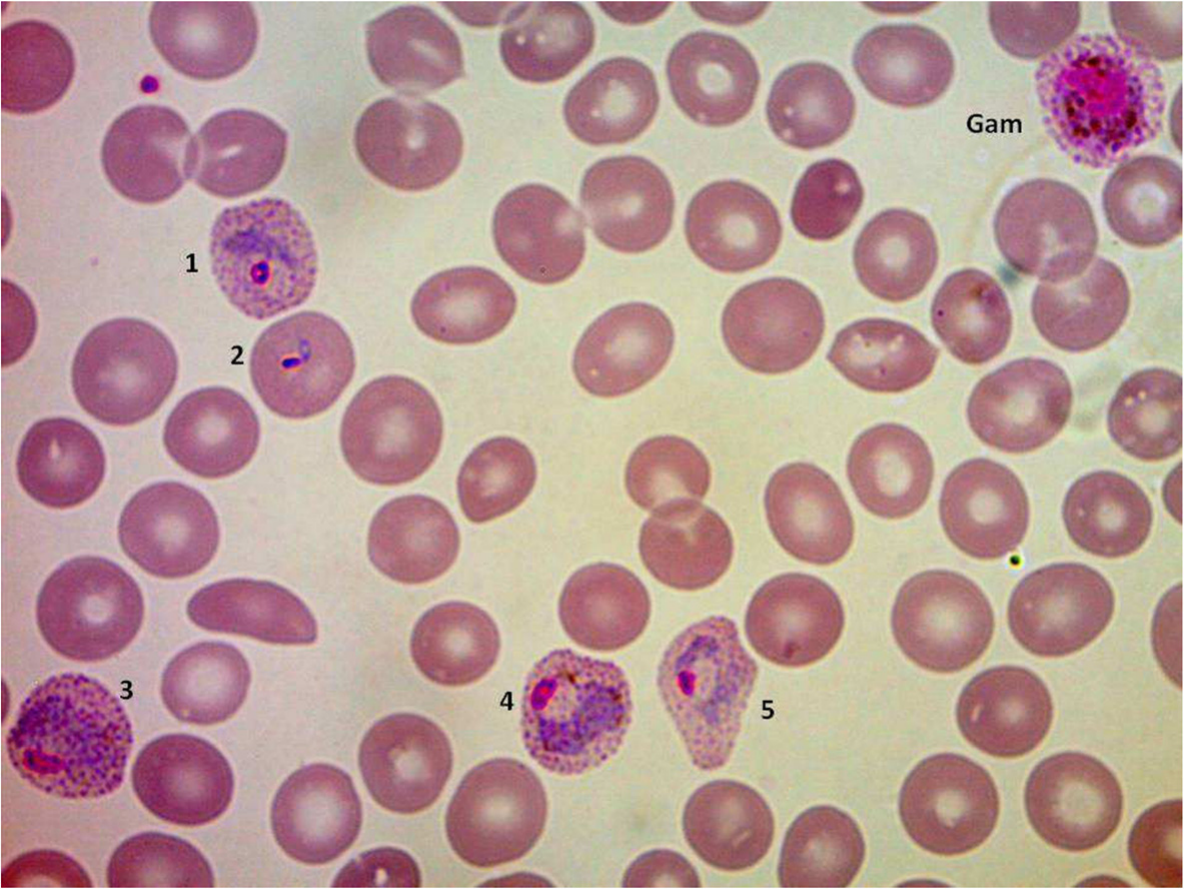
**Thin blood film of *Plasmodium vivax *case with 6.5%.** Morphological aspects are typical of *P. vivax*: throphozoites (blood red cells 1–5) and gametocyte (blood red cell with Gam). Five asexual forms for a total of 65 erythrocytes.

For *P. vivax*, it was 6.5%; Figure 2 shows the thin blood film; morphological aspects are typical of *P. vivax*. None of the 22 cases of *P. malariae* infection had a PC >1%.

**Figure 3:**
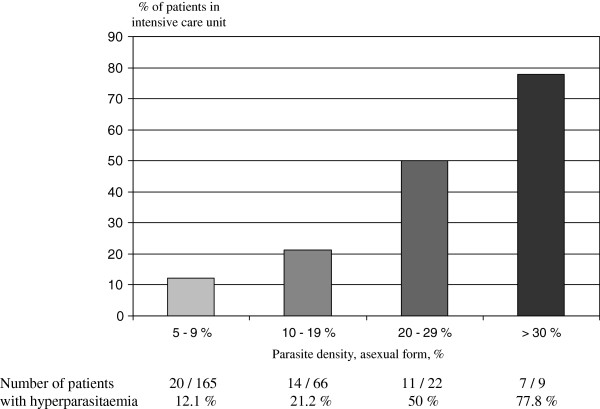
Hyperparasitaemia and hospitalization in an ICU (Cayenne Hospital).

The overall frequency of *P. falciparum* HP was 4.9% (252 + 7/4,840 + 417) and only 0.041% (3/6,897 + 417) for *P. vivax* HP (two orders of magnitude less frequent). Fifty-two of the 262 patients with HP (21.9%) were admitted to intensive care. The proportion increased steadily with CPL: 10.5% of cases (17/162) for parasitaemia of 4-9%; 21.2% (14/66) for parasitaemia of 10-19%; 50% (11/22) for parasitaemia of 20-29%; and, 77.8% (7/9) for parasitaemia ≥30% (Figure 3). The overall prevalence for falciparum malaria was 18.9%. The proportion of patients with HP who died was 11.8% (2/17) for HP <10% and 34.4% (11/32) for HP ≥10% (p ~ 0.08, Fisher test). In this study, malaria mortality was not assessed for non-HP (<4%) patient admitted to ICU.

## Discussion

**Figure 4:**
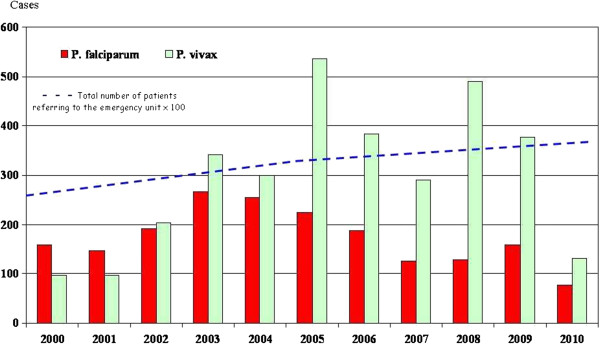
Bouts of malaria recorded in the Emergency Unit of Cayenne Hospital from 2000 to 2010.

Between 2000 and 2010 the distribution of Plasmodium species changed markedly over this period: *P. falciparum* predominated until 2001, with *P. vivax* subsequently becoming the major species, peaking in 2008 (two-thirds of cases) (Figure 4). This change has resulted from a large decrease in malaria incidence in western French Guiana, where *P. falciparum* predominated, and a substantial increase in the frequency of *P. vivax* malaria [6] in the eastern part of the country, although levels have subsequently decreased over the last two years [2].

*Plasmodium falciparum* in isolation accounts for 96.2% of HP cases, and even 98.9 % taking into account *P. falciparum/P. vivax* co-infections, where *P.falciparum* appears as the predominant species.

Therefore, only three cases of *P. vivax* alone were found, which accounts for 1.1%. These three cases were uncomplicated malaria attacks with no particular severity criteria and so no requiring admission in ICU. HP (>4%) is not a criterion for severity of non-falciparum malaria.

This paper does not deal with clinical or evolutionary aspects of HP, but confirms that this feature is a severity criterion for *falciparum* malaria in French Guiana. This long-standing view [7] is readily confirmed in cases of malaria with little or no immunity [8], as for the patients consulting at Cayenne.

The need for intensive care was correlated with parasite load but not mortality among HP patients (p=0.08). Two out of nine patients with a CPL ≥30% had undergone splenectomy [9] and one presented a major lupus flare-up treated with corticosteroids, patient with the highest parasitaemia (75%). These three patients survived without obvious sequelae despite having “microscopic” criteria for severity other than HP: presence of schizonts, some forming rosettes, a large proportion of mature trophozoites [10] and the presence of pigmented leukocytes [11]. These characteristics reflect a greater sequestered biomass, thus predicting a more severe disease.

The frequency of HP was higher in ICU patients. Over the last 11 years, the total number of bouts of *falciparum* malaria in French Guiana can be estimated at 15,400: mean of 3,500 cases per year for this period, according to the regional health agency of French Guiana (ARS French Guiana) × 11 years × 40/100 (% *P. falciparum*). For the same period, asexual form of *P. falciparum*, whatever the level of parasitaemia, was detected in 121 ICU patients immediately before or during the first days of their admission. Given the level of equipment and financial resources available in French Guiana, all patients requiring hospitalization in intensive care, particularly for severe malaria, were evacuated by road or by helicopter to CGH. In fact, ICU admission of a patient with malaria does not always correspond to a real severe malaria case according to WHO criteria. It should be noted that HP itself is not a criterion for intensive care hospitalization in French Guiana but it may influence the decision. Indeed the ICU admission for HP patients initially seen at Cayenne Hospital (the only hospital in French Guiana with ICU) is facilitated for reasons of prudence, the patient being on site. Without applying a correction factor, the theoretical frequency of ICU hospitalizations for all *falciparum* malaria acute cases (fever with asexual form of *P. falciparum* detected by microscopy) can be estimated in French Guiana as [121/15,400] = 0.8%. This is lower by a factor > 25 than that for cases of HP (21.9%).

This finding should not mask the absence of a clear link between PC based on peripheral blood screening and the severity of *falciparum* malaria in individual patients. There are several possible explanations. Circulating parasite load evaluation does not take into account the presence of mature stages in erythrocytes sequestered in deep capillaries (*P. falciparum*) and does not faithfully reflect the true parasite load. Furthermore, the proportion of circulating elements (visible and thus valuable) may vary considerably over time and with the parasite stage reached in bouts involving “synchronous” strains [12]. Note that the maximum parasitaemia value obtained for each malaria case was recorded. Peripheral parasitemia does not quantitative sequestered biomass. Plasma HRP2 level is a better indicator [13], but unfortunately not performed in Cayenne Hospital.

Other gravity criteria linked to receptivity and host immune defenses and to the virulence of strains other than their capacity to multiply must also be considered. In French Guiana the association of a specific *msp-1* allele (B-K1) with a specific *var* gene *(var-D)* was over-represented among patients with severe *versus* mild disease [14].

## Conclusions

In French Guiana during bouts of malaria, HP has been observed at a frequency of ~5% for *P. falciparum* and two orders of magnitude less frequent for *P. vivax*. Clearly high PC is a severity criterion for *falciparum* malaria in this endemic area. However, two of the patients with PC ≥30% were not admitted to the ICU and sequel-free cure in malaria patients with 75% parasitaemia is, therefore, possible.

## Abbreviations

CGH: Cayenne General Hospital; PC: Parasitic count; HP: Hyperparasitaemia; ICU: Intensive Care Unit.

## Competing interests

The author declare that they have no competing interests.

## Authors’ contributions

BC completed the design of the study, performed data analysis and interpretation and prepared the manuscript. MD participated in interpretation of data and manuscript revision. All authors read and approved the final manuscript.
